# Association between serum anion gap trajectory and mortality in hospitalized patients with sepsis: an analysis of the MIMIC-IV database

**DOI:** 10.3389/fendo.2025.1578078

**Published:** 2025-08-01

**Authors:** Lijuan Jing, Xiaopeng Shi, Lijun Xu, Xiangmei Zhao, Faliang Li, Lijie Qin

**Affiliations:** Department of Emergency Medicine, Henan Provincial People’s Hospital, Zhengzhou, China

**Keywords:** sepsis, anion gap, trajectory analysis, mortality, intensive care unit

## Abstract

**Background:**

Sepsis remains a leading cause of mortality in intensive care units (ICUs), with high morbidity and healthcare costs worldwide. The serum anion gap (AG), a marker of metabolic acidosis, has been associated with adverse outcomes in various critical illnesses. However, the prognostic value of longitudinal AG trajectories in sepsis remains underexplored. This study explored the link between dynamic AG trajectories and all-cause mortality in critically ill septic patients.

**Methods:**

A retrospective cohort study utilized data from the Medical Information Mart for Intensive Care IV (MIMIC-IV) database. Adult patients meeting Sepsis-3 criteria for sepsis were included. Group-based trajectory modeling was used to identify AG trajectories during the initial five days of ICU admission. Patients were classified into three trajectory groups: normal-level-stable trajectory (Class 1), high-level-decline trajectory (Class 2), and progressive acidosis trajectory (Class 3). Cox proportional hazards models evaluated the link between AG trajectories and ICU/hospital mortality, controlling for demographic, laboratory, and clinical severity factors. Subgroup and sensitivity analyses were performed to validate the findings.

**Results:**

Among 6,110 septic patients, three distinct AG trajectory groups were identified. Patients in Class 3 (decreasing high AG) had the highest mortality, with ICU mortality of 30.61% and hospital mortality of 35.85%, compared to Class 1 (ICU mortality: 14.46%, hospital mortality: 19.41%) and Class 2 (ICU mortality: 21.88%, hospital mortality: 31.88%). In fully adjusted models, Class 3 exhibited a significantly increased risk of ICU mortality [HR=1.72, (95% CI 1.43-2.07), P<0.001] and hospital mortality [HR=1.64, (95% CI 1.39-1.94), P<0.001] relative to Class 1. Subgroup analysis revealed a significant interaction between AG trajectories and heart failure status. Sensitivity analysis excluding patients with malignancies confirmed the robustness of the findings.

**Conclusion:**

Continuous monitoring of AG levels is crucial for risk assessment and personalized treatment, as rising AG levels significantly increase mortality risk. These findings underscore the potential of AG trajectories as a dynamic biomarker to improve sepsis management and patient outcomes.

## Introduction

Sepsis is a life-threatening syndrome of organ dysfunction caused by infection, with persistently high global incidence and mortality (1). Although the understanding of the pathophysiology of sepsis as a disease has gradually improved in recent years, sepsis remains a major threat to global health. The Global Burden of Disease Study reported that in 2017, there were about 48.9 million new sepsis cases globally, resulting in 11 million deaths, which constituted 19.7% of all global fatalities (2). A large sample study showed that the proportion of hospital-acquired sepsis was 23.6% of all hospital-treated sepsis cases, and 24.4% of cases of sepsis organ dysfunction were acquired during intensive care unit (ICU) hospitalization. Hospital-acquired sepsis is of major public health importance, and the burden on ICUs is particularly high (3, 4). In addition, sepsis is very expensive to treat. From 2009 to 2016, treating sepsis in the United States cost approximately $16,000 to $38,000 per patient, ranking it among the most expensive diseases to manage during that period (5). For patients and their families, the burden extends beyond the immediate financial strain, as they also face long-term physical and psychological challenges during the recovery process (6). Although in-hospital mortality rates for sepsis patients have significantly decreased from 25.7% in 2005 to 17.9% in 2019, the 28-day mortality rate for sepsis patients in the ICU remains over 30% (7, 8). Therefore, it is important to study the prognosis of severe sepsis.

The serum anion gap (AG) is a crucial metabolic indicator for evaluating electrolyte balance, particularly in acid-base disorders (9). Elevated AG typically indicates an increase in unmeasured anions, such as lactate, ketones, or other organic acids (10). Outside the context of sepsis, AG has been used to predict the risk of diseases like acute kidney injury, chronic obstructive pulmonary disease, and acute heart failure and has been associated with adverse outcomes (11–13). For instance, in patients with advanced chronic kidney disease, and a high anion gap (≥9.2 mmol/L) had 3.04 times the risk of renal failure replacement therapy and 5.56 times the risk of death compared to other patients (14). Similarly In patients undergoing CPR, the highest quartile of AG was associated with a 64% increased risk of in-hospital death, independent of traditional markers (15). However, research on AG in sepsis is relatively limited and mostly focuses on the significance of single measurement values (16, 17). These single-time-point data cannot fully reflect the complex pathophysiological changes in sepsis and fail to capture the dynamic changes in AG over time (18). In contrast, longitudinal trajectory analysis can more accurately depict the patterns of AG changes, thereby providing more detailed information for understanding the sepsis process. By monitoring the changes in AG over time, it is possible to better identify patients who may experience more severe complications or have a poorer prognosis.

This study examines the link between serum AG trajectories and all-cause mortality in sepsis patients using the Medical Information Mart for Intensive Care IV (MIMIC-IV) database. This not only helps improve existing risk stratification tools but also guides the selection of early interventions to improve the management strategies for sepsis patients and ultimately enhance their survival rates.

## Methods

### Research methodology and data origin

This retrospective cohort study analyzed data from the MIMIC-IV database, which includes de-identified health information of over 50,000 ICU patients at Beth Israel Deaconess Medical Center from 2008 to 2019 (19). The MIMIC-IV database includes comprehensive clinical data, such as demographics, vital signs, laboratory measurements, medications, and outcomes, making it suitable for investigating the association between longitudinal AG trajectories and mortality in critically ill septic patients. The Institutional Review Boards of Massachusetts Institute of Technology and Beth Israel Deaconess Medical Center approved the use of this database, waiving the need for informed consent due to data de-identification. One of the authors, Xiaopeng Shi, was granted access to this database (ID: 38652558).

### Study population

**Figure 1:**
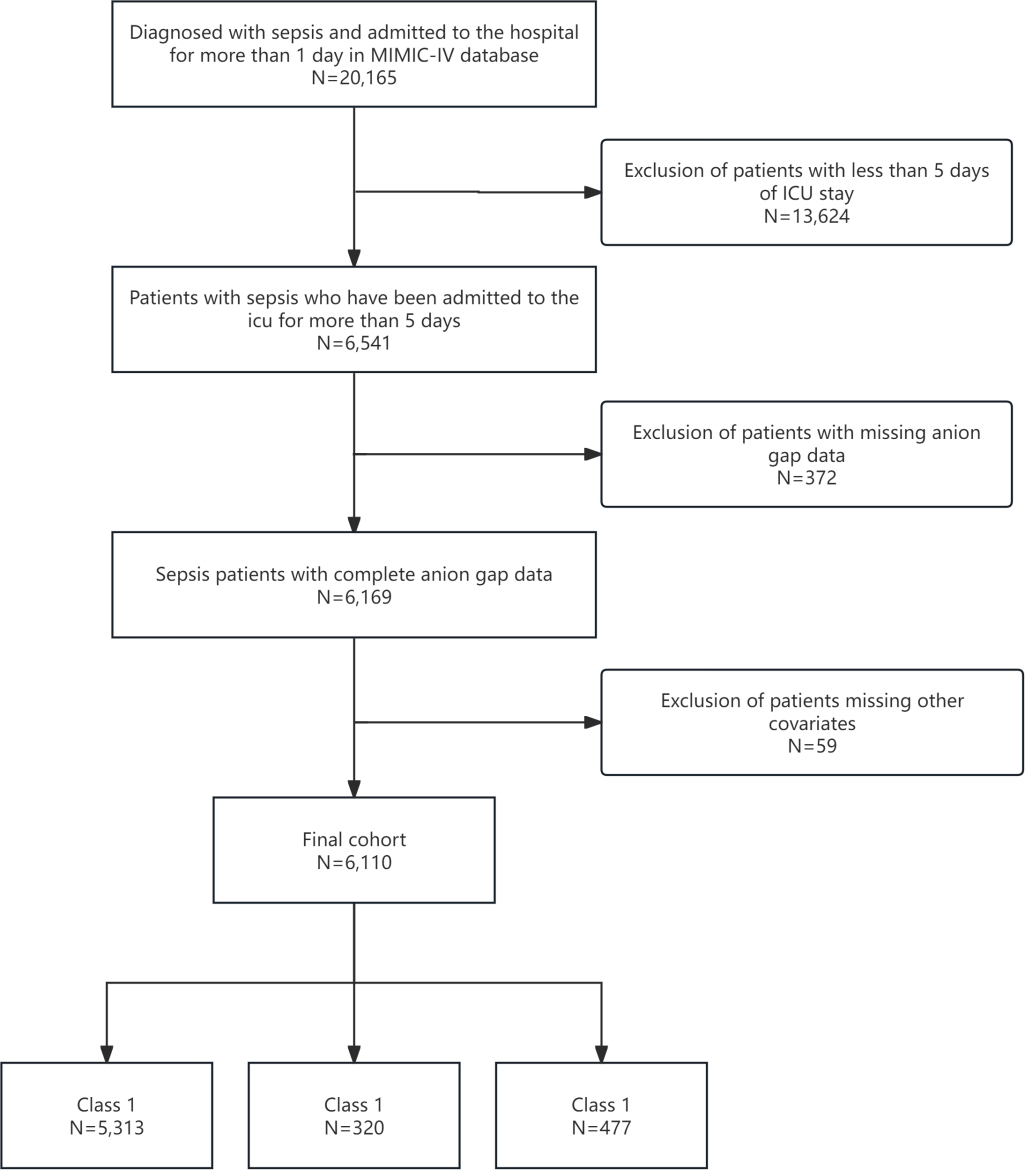
Flow diagram of the screening and enrollment of study participants.

We included adult patients (aged ≥18 years) diagnosed with sepsis based on Sepsis-3 criteria, which necessitate a suspected or confirmed infection and sequential organ failure assessment (SOFA) score increase of ≥2 points (20). Patients were excluded if they had an ICU stay of <5 days, missing AG measurements during the first five days, had fewer than two AG measurements, or had incomplete covariate data. From the MIMIC-IV database, 6,110 patients met the criteria and were stratified into three AG trajectory groups: Class 1 (n=5,313), Class 2 (n=320), and Class 3 (n=477). This ensured a robust cohort for longitudinal AG trajectory analysis (**Figure 1**).

### Data extraction and preprocessing

Relevant variables were extracted using Structured Query Language (SQL), encompassing demographic data (age, gender, and weight), laboratory results (serum AG, blood cell counts, electrolytes, glucose, urea nitrogen, and creatinine), vital signs (heart rate, respiratory rate, and blood pressure), and clinical severity scores [SOFA, simplified acute physiology score II (SAPS II), oxford acute severity of illness score (OASIS), and systemic inflammatory response syndrome (SIRS)].

AG was extracted for the first five days of ICU admission to capture the dynamic changes in AG over time. The latent class mixed model (LCMM) was used to identify the AG trajectory of the study population (21). This method allows for the identification of heterogeneous subgroups of patients based on their longitudinal AG measurements. Models with 1 to 5 trajectory categories were evaluated and the optimal number of categories was determined using the lowest value of the Akaike Information Criterion (AIC), the Bayesian Information Criterion (BIC), the Sample Adjusted BIC (SABIC), and the highest value of the entropy value (22). The main dependent variable was all-cause mortality, encompassing both ICU and hospital mortality. ICU mortality refers to death during the ICU stay, whereas hospital mortality encompasses death during the hospital stay, irrespective of discharge status.

### Statistical analysis

Baseline characteristics of the study population were compared across the three AG trajectory groups using descriptive statistics. Continuous variables were summarized as means ± standard deviation (SD) or medians with interquartile ranges (IQR) based on distribution and analyzed using Student’s t-test or the Mann-Whitney U test. Categorical variables were expressed as frequencies and percentages and analyzed using the chi-square or Fisher’s exact test.

Kaplan-Meier survival curves were created to illustrate survival differences among trajectory groups, with the log-rank test employed for comparing survival distributions. Cox proportional hazard models were used to assess the relationship between AG trajectories and mortality. Three models were developed: Model 1 (unadjusted), Model 2 (adjusted for demographic and laboratory variables), and Model 3 (Further adequate adjustment for clinical severity scores and comorbidities). The risk of ICU and hospital mortality for each AG trajectory group was evaluated using hazard ratios (HRs) and 95% confidence intervals (CIs). Subgroup analyses evaluated the consistency of the association between AG trajectories and mortality across various patient subgroups, including age, gender, and heart failure status. Interaction tests were performed to evaluate whether the association varied by subgroup. To ensure the robustness of the findings, sensitivity analyses were performed by excluding patients with malignancies.

Statistical analyses were conducted using R software (version 4.2.2), with a two-sided P-value of less than 0.05 deemed statistically significant.

## Results

### Determination of optimal AG trajectory classes

**Figure 2:**
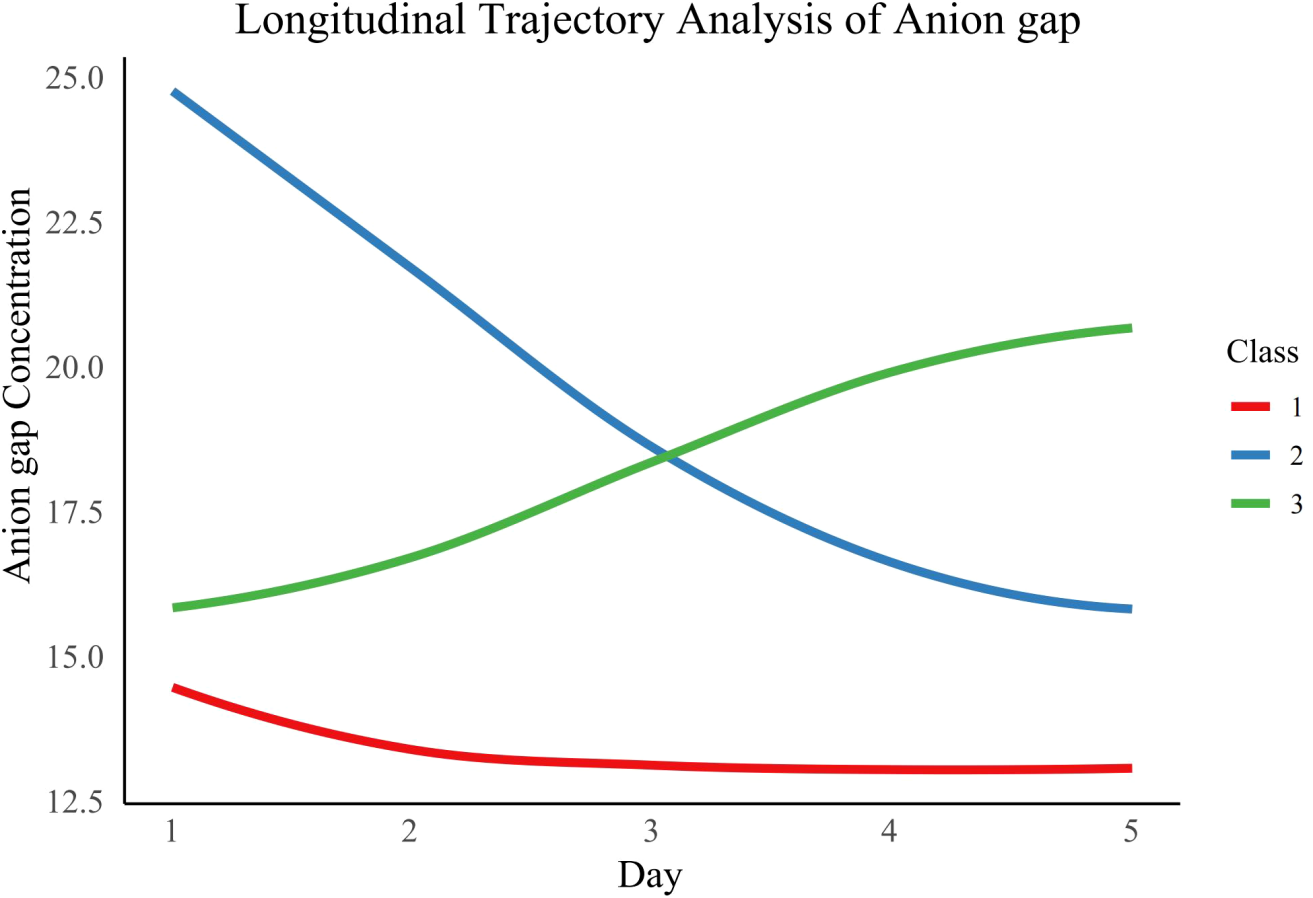
AG trajectory during the first 5 days in the ICU.

We assessed various statistical metrics, such as log-likelihood, AIC, BIC, SABIC, and entropy, to identify the best number of AG trajectory classes, ranging from 1 to 5 (**Supplementary Table S1**). A three-class model was chosen as the optimal solution due to its lowest AIC (149,412.5), BIC (149,479.7), and SABIC (149,448.0) values, an entropy of 0.8, and the condition that each class includes more than 5% of participants. The three classes were characterized as follows: Class 1 (normal-level-stable trajectory), Class 2 (high-level-decline trajectory), and Class 3 (progressive acidosis trajectory) (**Figure 2**). The average posterior probabilities for class membership were robust, with values of 0.9474 for Class 1, 0.8732 for Class 2, and 0.8129 for Class 3 (**Supplementary Table S2**).

### Baseline characteristics of patients

**Table 1** presents the baseline characteristics of 6,110 sepsis patients categorized by AG trajectory groups. The cohort’s average age was 64.80 ± 16.37 years, with age and gender distribution showing no significant variation among the trajectory groups. Notable variations were found in various laboratory parameters, such as white blood cell count (WBC), red blood cell count (RBC), platelet count, red blood cell distribution width (RDW), and levels of sodium, potassium, calcium, glucose, urea nitrogen, and creatinine. Notably, Compared with Class 1, patients in Class 2 and Class 3 had higher mean AG levels. Additionally, patients in Class 2 exhibited more severe organ dysfunction, as indicated by higher SOFA scores and SAPS II scores. In terms of mortality, Class 3 had the highest ICU mortality rate (30.61%) and hospital mortality rate (35.85%), followed by Class 2 with ICU mortality of 21.88% and hospital mortality of 31.88%, both significantly higher than Class 1 (ICU mortality 14.46%, hospital mortality 19.41%, P < 0.001 for both). Finally, **Supplementary Table S3** shows that there are differences between AGs with different trajectories within the first 5 days of ICU admission (P < 0.001).

**Table 1:** Baseline characteristics of patients stratified by AG trajectory groups.

Characteristics	All (n = 6110)	Class 1 (n = 5313)	Class 2 (n = 320)	Class 3 (n = 477)	*P*
Age, years	64.80 ± 16.37	64.76 ± 16.55	63.41 ± 14.48	66.11 ± 15.41	0.068
Male, n(%)	3520 (57.61)	3058 (57.56)	181 (56.56)	281 (58.91)	0.787
Weight, kg	84.95 ± 25.74	84.84 ± 25.67	84.12 ± 24.73	86.69 ± 27.18	0.272
WBC, K/µL	13.81 ± 10.64	13.68 ± 9.96	15.46 ± 11.77	14.22 ± 15.84	0.010
RBC, m/µL	3.59 ± 0.80	3.63 ± 0.79	3.39 ± 0.90	3.34 ± 0.77	<0.001
Platelet, K/µL	203.43 ± 111.14	206.87 ± 110.26	184.28 ± 132.02	177.94 ± 100.71	<0.001
RDW, %	15.17 ± 2.32	15.04 ± 2.24	16.37 ± 2.67	15.80 ± 2.64	<0.001
Sodium, mmol/L	138.63 ± 5.74	138.78 ± 5.63	137.94 ± 7.31	137.42 ± 5.61	<0.001
Potassium, mmol/L	4.22 ± 0.79	4.19 ± 0.77	4.64 ± 0.97	4.34 ± 0.81	<0.001
Calcium, mg/dL	8.19 ± 0.95	8.20 ± 0.93	7.98 ± 1.16	8.19 ± 1.05	<0.001
Glucose, mg/dL	155.62 ± 79.07	155.08 ± 77.37	173.15 ± 108.09	149.90 ± 73.19	<0.001
Urea nitrogen, mg/dL	29.33 ± 24.19	26.61 ± 20.61	61.28 ± 41.90	38.11 ± 27.33	<0.001
Creatinine, mg/dL	1.56 ± 1.58	1.35 ± 1.18	4.08 ± 3.28	2.23 ± 2.03	<0.001
SOFA	6.74 ± 3.91	6.34 ± 3.70	11.00 ± 4.09	8.31 ± 3.98	<0.001
SAPS II	42.95 ± 14.57	41.78 ± 14.06	56.40 ± 14.91	46.92 ± 14.72	<0.001
OASIS	36.55 ± 8.37	36.20 ± 8.21	41.22 ± 9.12	37.27 ± 8.59	<0.001
SIRS	2.92 ± 0.89	2.92 ± 0.90	3.12 ± 0.81	2.83 ± 0.89	<0.001
Heart rate, minute	92.12 ± 21.47	91.70 ± 21.37	99.85 ± 22.16	91.52 ± 21.11	<0.001
Respiratory rate, minute	20.19 ± 6.53	20.04 ± 6.54	22.42 ± 6.31	20.25 ± 6.30	<0.001
Hypertension, n (%)	2547 (41.69)	2319 (43.65)	89 (27.81)	139 (29.14)	<0.001
DM, n (%)	1682 (27.53)	1402 (26.39)	107 (33.44)	173 (36.27)	<0.001
Heart failure, n (%)	1881 (30.79)	1583 (29.79)	118 (36.88)	180 (37.74)	<0.001
MI, n (%)	510 (8.35)	411 (7.74)	47 (14.69)	52 (10.90)	<0.001
Malignant tumors, n (%)	845 (13.83)	728 (13.70)	43 (13.44)	74 (15.51)	0.536
Stroke, n (%)	651 (10.65)	581 (10.94)	34 (10.62)	36 (7.55)	0.071
ICU mortality, n (%)	984 (16.10)	768 (14.46)	70 (21.88)	146 (30.61)	<0.001
Hospital mortality, n (%)	1304 (21.34)	1031 (19.41)	102 (31.88)	171 (35.85)	<0.001

*WBC* white blood cell; *RBC* red blood cell; *RDW* red blood cell distribution width; *SOFA* sequential organ failure assessment; *SAPS II* simplified acute physiology score II; *OASIS* oxford acute severity of illness score; *SIRS* systemic inflammatory response syndrome; *DM* diabetes mellitus; *MI* myocardial infarction; *ICU* intensive care unit.

Continuous variables are expressed as mean ± standard deviation, and categorical variables are expressed as n (%).

### Association between AG trajectories and mortality

**Figure 3:**
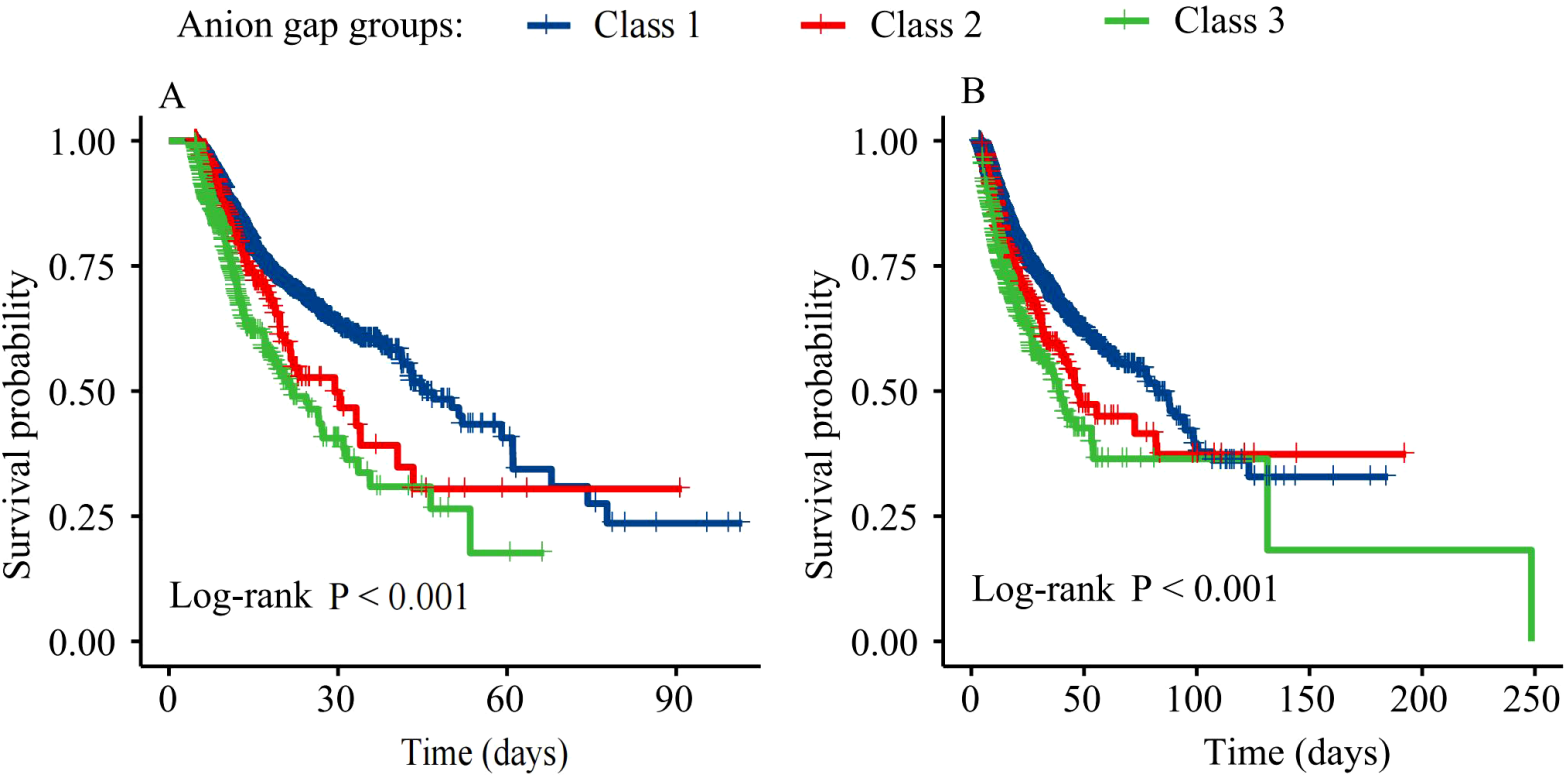
Kaplan-Meier survival estimates for ICU **(A)** and hospital **(B)** mortality by AG trajectory.

**Figure 3** presents Kaplan-Meier survival curves for ICU and hospital mortality. Survival rates significantly differed among the three AG trajectory groups for both ICU and hospital mortality (log-rank P<0.001). Class 3 patients had the lowest survival rates, followed by Class 2 and Class 1.

Cox proportional hazard models were used to assess the relationship between AG trajectories and mortality (**Table 2**). In the unadjusted model (Model 1), patients in Class 2 and Class 3 exhibited significantly elevated risks of both ICU mortality [Class 2: HR=1.36 (95% CI 1.07-1.74), P=0.013; Class 3: HR=2.04 (95% CI 1.71-2.44), P<0.001] and hospital mortality [Class 2: HR=1.36 (95% CI 1.11-1.67), P=0.003; Class 3: HR=1.85 (95% CI 1.58-2.18), P<0.001] compared to Class 1. Even after controlling demographic and laboratory variables (Model 2), Class 3 showed a significant association with ICU mortality [HR=1.87, (95% CI 1.56-2.24), P<0.001] and hospital mortality [HR=1.70, (95% CI 1.44-2.00), P<0.001]. In Model 3, after further adjusting clinical severity scores and comorbidities, Class 3 patients exhibited a significantly increased risk of ICU mortality [HR=1.72, (95% CI 1.43-2.07), P<0.001] and hospital mortality [HR=1.64, (95% CI 1.39-1.94), P<0.001]. In contrast, the risk of death in the ICU and during hospitalization for class 2 patients was not significantly different from that of class 1 patients (P>0.05).

**Table 2:** Relationship between AG trajectories and mortality in patients with sepsis in different models.

AG trajectory	Model 1		Model 2		Model 3	
	HR (95%CI)	*P*	HR (95%CI)	*P*	HR (95%CI)	*P*
ICU mortality						
Class 1	1.00		1.00		1.00	
Class 2	1.36 (1.07, 1.74)	0.013	1.18 (0.92, 1.51)	0.199	0.89 (0.68, 1.17)	0.407
Class 3	2.04 (1.71, 2.44)	<0.001	1.87 (1.56, 2.24)	<0.001	1.72 (1.43, 2.07)	<0.001
Hospital mortality						
Class 1	1.00		1.00		1.00	
Class 2	1.36 (1.11, 1.67)	0.003	1.28 (1.04, 1.57)	0.019	1.00 (0.80, 1.26)	0.988
Class 3	1.85 (1.58, 2.18)	<0.001	1.70 (1.44, 2.00)	<0.001	1.64 (1.39, 1.94)	<0.001

Model 1: Crude.

Model 2: Adjust for age, gender, weight, WBC, RBC, and RDW.

Model 3: Adjust for age, gender, weight, WBC, RBC, platelet, RDW, sodium, potassium, calcium, glucose, urea nitrogen, creatinine, SOFA score, SAPS II score, OASIS score, SIRS score, heart rate, respiratory rate, hypertension, DM, heart failure, and MI.

### Subgroup analysis and interaction effect

Subgroup analyses were performed to evaluate the consistency of the association between AG trajectory and mortality across different patient subgroups (**Table 3**). The results indicated that the relationship between AG trajectories and mortality was generally consistent across most subgroups. However, a significant interaction was observed between the presence or absence of heart failure and AG trajectory in the analysis of both ICU mortality and hospital mortality (P for interaction = 0.047 for ICU mortality and 0.012 for hospital mortality).

**Table 3:** The relationship between serum AG trajectories and mortality in patients with sepsis in different subgroups.

Characteristics	Class 1	Class 2	Class 3	P for interaction
ICU mortality				
Age				0.595
≥70	1.00	0.82 (0.59, 1.16)	1.70 (1.35, 2.14)	
<70	1.00	1.07 (0.67, 1.69)	1.90 (1.38, 2.63)	
Gender				0.271
female	1.00	0.77 (0.51, 1.16)	2.16 (1.62, 2.86)	
male	1.00	0.98 (0.67, 1.42)	1.58 (1.23, 2.04)	
Hypertension				0.116
no	1.00	0.72 (0.51, 1.02)	1.71 (1.37, 2.14)	
yes	1.00	1.20 (0.76, 1.89)	1.93 (1.38, 2.71)	
Heart failure				0.047
no	1.00	1.18 (0.84, 1.65)	1.78 (1.39, 2.28)	
yes	1.00	0.55 (0.34, 0.90)	1.74 (1.31, 2.31)	
MI				0.735
no	1.00	0.88 (0.65, 1.18)	1.84 (1.50, 2.24)	
yes	1.00	0.63 (0.31, 1.28)	1.34 (0.77, 2.35)	
DM				0.927
no	1.00	0.89 (0.64, 1.23)	1.72 (1.37, 2.17)	
yes	1.00	0.82 (0.50, 1.36)	1.96 (1.42, 2.71)	
Hospital mortality				
Age				0.139
≥65	1.00	0.89 (0.67, 1.19)	1.56 (1.26, 1.92)	
<65	1.00	1.26 (0.86, 1.83)	1.90 (1.42, 2.56)	
Gender				0.303
female	1.00	0.82 (0.58, 1.16)	1.68 (1.30, 2.18)	
male	1.00	1.16 (0.85, 1.57)	1.71 (1.36, 2.14)	
Hypertension				0.715
no	1.00	0.93 (0.71, 1.23)	1.64 (1.34, 2.02)	
yes	1.00	1.02 (0.67, 1.55)	1.83 (1.35, 2.50)	
Heart failure				0.012
no	1.00	1.27 (0.96, 1.68)	1.62 (1.29, 2.03)	
yes	1.00	0.59 (0.39, 0.90)	1.77 (1.37, 2.29)	
MI				0.865
no	1.00	0.97 (0.76, 1.24)	1.71 (1.43, 2.05)	
yes	1.00	0.79 (0.42, 1.48)	1.40 (0.84, 2.33)	
DM				0.969
no	1.00	1.02 (0.78, 1.35)	1.70 (1.38, 2.09)	
yes	1.00	0.88 (0.58, 1.34)	1.71 (1.27, 2.29)	

Both subgroup analyses and interaction tests were adjusted for covariates that appeared in Model 3.

### Sensitivity analysis

We conducted a sensitivity analysis by omitting patients with malignancies to verify the robustness of our findings (**Supplementary Table S4**). In the fully adjusted Model 3, Class 3 patients exhibited significantly elevated risks of ICU mortality [HR=1.90, (95% CI 1.55-2.32), P<0.001] and hospital mortality [HR=1.78, (95% CI 1.48-2.14), P<0.001], aligned with the primary analysis. Class 2 patients demonstrated no significant link to mortality, with ICU mortality [HR=0.90, (95% CI 0.67-1.21), P=0.468] and hospital mortality [HR=1.02, (95% CI 0.79-1.31), P=0.892] both showing non-significant associations.

## Discussion

This study examined the association between longitudinal AG trajectories and mortality in critically ill sepsis patients. We identified three AG trajectory patterns: normal-level-stable trajectory (Class 1), high-level-decline trajectory (Class 2), and progressive acidosis trajectory (Class 3). Class 3 patients exhibited significantly higher mortality risks in both the ICU and the hospital. Class 2 patients exhibited no significant difference in mortality compared to those with Class 1. Additionally, we found that the presence of heart failure mediated the association between AG trajectories and mortality. These findings underscore the importance of dynamic AG monitoring in sepsis management, offering a potential tool for early risk assessment and personalized treatment approaches.

Recent research has increasingly emphasized utilizing longitudinal biomarker trajectories for predicting sepsis outcomes. Lactate is an important biomarker for assessing tissue perfusion and oxygenation in patients with sepsis. Studies have shown that the lactate trajectory is closely associated with mortality. Similar to the AG trajectory, a sustained rise in lactate levels also indicates a poor prognosis, while a decrease in lactate levels suggests a positive response to treatment (23). CRP is a key biomarker for assessing inflammatory responses. Studies have shown that the trajectory of CRP levels is closely linked to mortality in sepsis patients. Similar to the AG trajectory, a sustained rise in CRP levels indicates a poor prognosis, while a decrease in CRP levels suggests effective control of the inflammatory response (24). Although AG, lactate, and CRP are all important biomarkers, the AG trajectory provides unique insights. Therefore, the AG trajectory can complement the lactate and CRP trajectories, providing a more comprehensive picture of the patient’s pathophysiology.

An increase in AG levels usually reflects the presence of metabolic acidosis, which may be due to tissue hypoperfusion, increased anaerobic metabolism, or accumulation of unmeasured anions (such as lactate, ketone bodies, or other organic acids) due to renal insufficiency (25–28). In class 3 (progressive acidosis trajectory) patients, AG levels continued to rise, indicating the persistence of metabolic acidosis and tissue hypoperfusion, which may be due to inadequate resuscitation or uncontrolled persistent infection (29, 30). Patients in Class 2 initially exhibited higher AG levels upon admission to the ICU, but these levels significantly decreased over the following days. This change may reflect a positive response to treatment, such as through effective fluid resuscitation, the use of vasoactive drugs, or other therapeutic measures, which successfully improve tissue perfusion and metabolic status. The decline in AG levels suggests that the patient’s metabolic acidosis has been effectively corrected, and the tissue hypoperfusion state has been improved, thereby reducing the risk of death. Compared to Class 3 (progressive acidosis trajectory), patients with Class 2 show better physiological recovery during treatment, highlighting the importance of dynamic monitoring of AG levels. This can help clinicians promptly identify patients who do not respond well to treatment and take more proactive intervention measures. In clinical practice, patients with Class 2 may require closer monitoring to ensure that the decline in AG levels is sustained and there are no other complications. This may involve regular blood gas analysis, electrolyte monitoring, and organ function assessments.

Longitudinal trajectories, on the other hand, allow for the identification of patterns associated with recovery or deterioration, enabling clinicians to tailor interventions based on real-time trends (31). In our study, we utilized repeated AGs to capture the dynamic nature of metabolic disturbances in sepsis. This approach aligns with emerging evidence that longitudinal biomarker trajectories offer superior prognostic information compared to static measurements (32). Our findings suggest increased ICU and in-hospital mortality in patients with progressive acidosis trajectory (Class 3) [compared with normal-level-stable trajectory (Class 1)], but this relationship was not observed in the high-level-decline trajectory (Class 2). Our study enhances existing research by highlighting the clinical significance of AG trajectories in sepsis prognosis, especially for identifying patients with the highest mortality risk. The trajectory of dynamic biomarkers provides richer information than measurements taken at a single point in time, allowing for a more accurate reflection of disease progression and treatment response. Integrating AG trajectories with other biomarker trajectories, such as lactate and CRP, can enhance the predictive power for the prognosis of sepsis patients. Future research could explore integrating AG trajectories into existing predictive models, such as the SOFA score and SAPS II score. Through multivariate analysis, the independent contribution of AG trajectories to predictive models can be assessed, and the model’s predictive performance can be optimized. Dynamic monitoring of AG trajectories can provide clinicians with real-time assessments of the patient’s condition, enabling timely adjustments to the treatment plan. For instance, patients with continuously rising AG levels may require more aggressive resuscitation measures or further infection control.

This study has certain limitations. The study’s reliance on a single database may restrict the generalizability of its findings. Second, while we adjusted for numerous confounders, residual confounding from unmeasured variables cannot be ruled out. Third, the study focused on AG trajectories during the first five days of ICU admission, and longer-term trajectories were not assessed. Observational design limits causal inferences, necessitating further prospective studies to validate our findings. This study offers important insights into the prognostic value of AG trajectories in sepsis, emphasizing their potential as a dynamic biomarker for risk stratification and clinical decision-making, despite certain limitations.

## Conclusion

This study suggests that an increase in serum AG over time is associated with an increased risk of death in patients with severe sepsis. Overall, serum AG trajectory may be a practical and direct predictor of prognosis in patients with sepsis.

## Ethical approval

No additional informed consent was required as de-identified data was used and the data was sourced from the ethically approved MIMIC database.

## Data Availability

The raw data supporting the conclusions of this article will be made available by the authors, without undue reservation.
