# Supplementary material for: Association between serum anion gap trajectory and mortality in hospitalized patients with sepsis: an analysis of the MIMIC-IV database
Source: Front Endocrinol (Lausanne). 2025 Aug 1;16:1578078. doi: 10.3389/fendo.2025.1578078 (PMC12353743; doi:10.3389/fendo.2025.1578078)
Supplement: Supplementary file 1 [file Table1.docx]

**Supplementary Material**

**Table S1.** Metrics for determining the optimal number of classes.

**Table S2.** MIMIC-IV average posterior probabilities.

**Table S 3.** AG size and comparison per day within each trajectory.

**Table S 4.** Relationship between AG trajectories and mortality in different models after removing septic patients with comorbid malignancies.

| **Table S1.** Metrics for determining the optimal number of classes. | | | | | | | | | | |
| --- | --- | --- | --- | --- | --- | --- | --- | --- | --- | --- |
| **No.of classes** | **Log likelihood** | **AIC** | **BIC** | **SABIC** | **Entropy** | **Proportion of participants (%)** | | | | |
|  |  |  |  |  |  | **Class 1** | **Class 2** | **Class 3** | **Class 4** | **Class 5** |
| 1 | -76081.8 | 152171.6 | 152198.5 | 152185.8 | 1 | 100 |  |  |  |  |
| 2 | -75264.6 | 150543.2 | 150590.3 | 150568.0 | 0.9 | 5.6 | 94.4 |  |  |  |
| 3 | -74696.2 | 149412.5 | 149479.7 | 149448.0 | 0.8 | 87.0 | 5.2 | 7.8 |  |  |
| 4 | -74543.4 | 149112.8 | 149200.2 | 149158.9 | 0.9 | 0.6 | 6.6 | 84.7 | 8.1 |  |
| 5 | -74396.1 | 148824.2 | 148931.8 | 148881.0 | 0.9 | 5.6 | 83.0 | 7.0 | 0.6 | 3.9 |
| *AIC* akaike information criterion, *BIC* bayesian information criteria, *SABIC* sample-adjusted information criteria. | | | | | | | | | | |

| **Table S2.** MIMIC-IV average posterior probabilities. | | | |
| --- | --- | --- | --- |
|  | **Prob 1** | **Prob 2** | **Prob 3** |
| Class 1 | **0.9474** | 0.0137 | 0.0389 |
| Class 2 | 0.1242 | **0.8732** | 0.0025 |
| Class 3 | 0.1844 | 0.0027 | **0.8129** |

| **Table S 3.** AG size and comparison per day within each trajectory. | | | | | |
| --- | --- | --- | --- | --- | --- |
|  | **All (n = 6110)** | **Class 1 (n = 5313)** | **Class 2 (n = 320)** | **Class 3 (n = 477)** | ***P*** |
|  |  |  |  |  |  |
| Day 1 AG | 15.10 ± 3.99 | 14.45 ± 3.06 | 24.75 ± 4.55 | 15.83 ± 4.19 | <.001 |
| Day 2 AG | 14.07 ± 3.85 | 13.38 ± 3.13 | 21.67 ± 4.52 | 16.71 ± 3.98 | <.001 |
| Day 3 AG | 13.81 ± 3.75 | 13.11 ± 3.13 | 18.57 ± 4.47 | 18.34 ± 4.02 | <.001 |
| Day 4 AG | 13.75 ± 3.68 | 13.03 ± 2.99 | 16.60 ± 4.13 | 19.89 ± 3.73 | <.001 |
| Day 5 AG | 13.79 ± 3.64 | 13.05 ± 2.88 | 15.82 ± 3.93 | 20.67 ± 3.41 | <.001 |
| Continuous variables are expressed as mean ± standard deviation | | | | | |

| **Table S 4.** Relationship between AG trajectories and mortality in different models after removing septic patients with comorbid malignancies. | | | | | | |
| --- | --- | --- | --- | --- | --- | --- |
|  | **Model 1** | | **Model 2** | | **Model 3** | |
|  | **HR**  **(95%CI)** | ***P*** | **HR (95%CI)** | ***P*** | **HR (95%CI)** | ***P*** |
| ICU mortality | | | | | | |
| Anion gap trajectory |  |  |  |  |  |  |
| Class 1 | 1.00 |  | 1.00 |  | 1.00 |  |
| Class 2 | 1.39 (1.07, 1.81) | 0.015 | 1.19 (0.91, 1.56) | 0.204 | 0.90 (0.67, 1.21) | 0.468 |
| Class 3 | 2.20 (1.81, 2.66) | <0.001 | 2.05 (1.68, 2.49) | <0.001 | 1.90 (1.55, 2.32) | <0.001 |
| Hospital mortality | | | | | | |
| Anion gap trajectory |  |  |  |  |  |  |
| Class 1 | 1.00 |  | 1.00 |  | 1.00 |  |
| Class 2 | 1.41 (1.13, 1.76) | 0.002 | 1.34 (1.07, 1.67) | 0.011 | 1.02 (0.79, 1.31) | 0.892 |
| Class 3 | 1.99 (1.67, 2.38) | <0.001 | 1.84 (1.54, 2.20) | <0.001 | 1.78 (1.48, 2.14) | <0.001 |
| Model1: Crude. | | | | | | |
| Model2: Adjust for age, gender, weight, WBC, RBC, and RDW. | | | | | | |
| Model3: Adjust for age, gender, weight, WBC, RBC, platelet, RDW, sodium, potassium, calcium, glucose, urea nitrogen, creatinine, SOFA score, SAPS II score, OASIS score, SIRS score, heart rate, respiratory rate, hypertension, DM, heart failure, and MI. | | | | | | |
